# Association of Nitrate, Nitrite, and Total Organic Carbon (TOC) in Drinking Water and Gastrointestinal Disease

**DOI:** 10.1155/2013/603468

**Published:** 2013-04-15

**Authors:** Samaneh Khademikia, Zahra Rafiee, Mohammad Mehdi Amin, Parinaz Poursafa, Marjan Mansourian, Amir Modaberi

**Affiliations:** ^1^Department of Environmental Health Engineering, Isfahan University of Medical Sciences, Isfahan 81676-36954, Iran; ^2^Environment Research Center, Isfahan University of Medical Sciences, Isfahan 81676-36954, Iran; ^3^School of Public Health, Isfahan University of Medical Sciences, Isfahan 81676-36954, Iran; ^4^Natural Resources Department, Lorestan University, Lorestan, Iran

## Abstract

*Objective*. We aimed to investigate the amounts of nitrate, nitrite, and total organic carbon (TOC) in two drinking water sources and their relationship with some gastrointestinal diseases. *Methods*. This cross-sectional study was conducted in 2012 in Iran. Two wells located in residential areas were selected for sampling and measuring the TOC, nitrate (NO3^−^), and nitrite (NO2^−^). This water is used for drinking as well as for industrial and agricultural consumption. Nitrate and nitrite concentrations of water samples were analyzed using DR 5000 spectrophotometer. The information of patients was collected from the records of the main referral hospital of the region for gastrointestinal diseases. *Results*. In both areas under study, the mean water nitrate and nitrite concentrations were higher in July than in other months. The mean TOC concentrations in areas 1 and 2 were 2.29 ± 0.012 and 2.03 ± 0.309, respectively. Pollutant concentration and gastrointestinal disease did not show any significant relationship (*P* > 0.05). *Conclusion*. Although we did not document significant association of nitrite, nitrate, and TOC content of water with gastrointestinal diseases, it should be considered that such health hazards may develop over time, and the quality of water content should be controlled to prevent different diseases.

## 1. Introduction

Nitrate is considered as the most prevalent chemical contaminant in the world's groundwater. Organic and inorganic sources of nitrogen are converted to nitrate. After reducing, nitrate can be biologically transformed to nitrogen gas. The growing contamination of public and private well drinking water by nitrate is mostly because of the widespread use of commercial fertilizers and waste [1].

Groundwater is used for agricultural and industrial consumption as well as for drinking water. Humans have altered the nitrogen cycle dramatically over the last decades, and as a result, nitrate is increasingly accumulating in water resources. Globally, human nitrogen production has increased significantly since 1950 due to the use of nitrogen fertilizers. In agricultural areas, groundwater and private and low depth wells have higher levels of nitrate. Fertilizers are most important contributing factor in agricultural areas; however, nitrogen from human waste seems to be an essential source in urban areas with deficient centralized water and sanitation systems [2].

Nitrate is in solution form and is mobile. It could become spread in groundwater and is one of the most common pollutant and concern for human health. It may have several health hazards. Drinking water contamination with nitrate could increase cancer risk, because nitrate could reduce to nitrite and the following nitrosation reactions give rise to N-nitroso compounds, which are especially carcinogenic and can act systemically. The suggested regulatory limit for nitrate is too conservative. A symposium in 2004 on drinking water nitrate and health assessed nitrate exposures and related health effects about the current regulatory limit. The contribution of drinking water nitrate toward endogenous formation of N-nitroso compounds was evaluated with a focus toward identifying subpopulations with increased rates of nitrosation [3, 4].

Drinking nitrate and nitrite under conditions likely to form N-nitroso compounds (NOCs), called endogenous nitrosation, is considered possibly carcinogenic to humans. Nitrate in drinking water is completely related to urine nitrate levels as well as excretion of nitrosoproline, a biomarker of endogenous nitrosation [4].

Water with high nitrate concentration is not suitable for human consumption, especially when its concentration exceeded the threshold limit (50 mg/L) recommended by the health authorities such as the World Health Organization (WHO) [5].

Human alteration of the nitrogen cycle has resulted in continuing accumulation of nitrate in the water resources. According to recent studies, a significant relationship exists between nitrate in drinking water and cancer types [3, 5–7].

Chemical analysis of a study in Iran showed that groundwater and water supply have considerably high levels of nitrate (from 52.3 mg/L to 52.69 mg/L). Nitrate level in some underground water wells in the landfill area was over the drinking water standard (50 mg per liter) [8, 9].

Growing body of evidence proposes the relationship between nitrate level in drinking water and gastric cancer [10–12].

This study aimed to investigate the amounts of nitrate, nitrite, and TOC in two drinking water sources and the relationship of nitrate and nitrite levels with some gastrointestinal diseases.

## 2. Materials and Methods

This cross-sectional study was conducted from Feb to June 2012 in Shahrekord city in central part of Iran. We selected this city because the level of nitrate in drinking water wells is high in this area with many underground water sources including approximately 493 deep wells, 321 wells, 171 deep semisubterranean, and 93 springs.

Two wells located in residential areas were selected for sampling and measuring TOC, nitrate (NO3^−^), and nitrite (NO2^−^). This water is used for drinking as well as for industrial and agricultural consumption.

Since the concentration of nitrate is usually constant year-round, we limited our study time to 5 months to save time and costs.

To determine the amount of nitrate and nitrite from these two wells, we collected a total of 18 samples (9 samples per well) and six samples (three samples per well) for TOC test.

### 2.1. Water Sampling

For sampling, we used one-liter plastic containers and filled each bottle with water of wells; then we emptied out their contents and once more about 900 mL of water was filled up to provide enough space for better shaking and mixing.

Samples were delivered to the Chemistry Laboratory of Health Faculty, Isfahan University of Medical Sciences, in less than 3 hours, and then they were analyzed after 24 hours.

Nitrate and nitrite concentrations of samples were read and analyzed using DR 5000 spectrophotometer with a wavelength of 220–275 nm. Experiments were repeated two times for each sample.

### 2.2. Using Spectrophotometer

The spectrophotometer uses indicator compounds to determine the concentration of nitrate in samples. The HACH Test kit was used; it involves mixing a premeasured amount of the indicator compound with a 50 mL of sample water; then the absorption in a particular wavelength was measured. After mixing and a delay for reaction, a color appeared (for nitrogen it is amber color), the darkness of which was measured in the spectrophotometer. The sample was introduced to the spectrophotometer via a pour-through-cell apparatus to minimize optical interferences from sample handling.

The program offered by the company (HACH 357 N Nitrate UV) was selected from the menu on the device. The device can be calibrated with distilled water. 50 mL of sample prepared with 1 mL of 1 N hydrochloric acid and the prepared sample was transferred to the quartz cell. Thereafter, the absorbance was displayed and we read it.

### 2.3. Measuring Nitrite

Measuring nitrite schedule was provided by the spectrophotometer (371 N Nitrite LR PP). This device was calibrated with samples of water, then we added a package of NitriVer 3 Nitrite reagent to 10 mL water samples, and after 20 minutes we read the absorption rate.

### 2.4. Measuring TOC

For measuring TOC, samples were transferred to laboratory of Isfahan Water and Sewage Company.

### 2.5. Gastrointestinal Diseases Hospital Records

The information of patients was collected from the Medical Records Department of Gastroenterology of Al-Zahra Hospital, Isfahan University of Medical Sciences. Patients with gastrointestinal diseases were referred from Shahrekord to this hospital. We selected this hospital because it is the main referral hospital of the region for gastrointestinal diseases, and patients of neighbor cities, including Shahrekord, are referred to this center.

### 2.6. Statistical Analysis

For statistical analysis, we categorized diseases to the following four groups: (1) bile duct diseases; (2) gastric diseases; (3) intestinal diseases; (4) other gastrointestinal diseases.

Data were analyzed by the Statistical Package for Social Sciences software version 20.0 (SPSS Inc, Chicago, IL, USA). Data are presented as mean and standard deviation. Comparison of values in various groups was assessed by One-Sample *t*- and Kruskall Wallis tests. The significance level was set at *P* < 0.05.

## 3. Results

The results of the analysis of water samples for nitrate, nitrite and TOC measurements are presented in Table 1. Results of descriptive analysis showed that the mean of nitrate and nitrite concentrations obtained in two areas was higher in July 2012 than in other months.

The mean of TOC concentrations in areas 1 and 2 was 2.29 ± 0.012 and 2.03 ± 0.309, respectively (*P* > 0.05).

The frequency of abovementioned diseases according to the codes ((1) bile duct diseases; (2) gastric diseases; (3) intestinal diseases; (4) other gastrointestinal diseases) was as follows: 47.6%, 19%, 19%, and 14.3%.

The corresponding numbers in two sampling areas were as follows:

 area 1: 0.25%, 0 %, 50%, and 25%,
 area 2: 52.9%, 23.5%, 11.8%, and 11.8%.

Evaluation of the relationship between pollutant concentration and gastrointestinal disease did not show any significant relationship in two cities (*P* > 0.05).

## 4. Discussion

This study aimed to evaluate the association of nitrite, nitrate, and TOC in water of two areas with the frequency of some common gastrointestinal diseases. We did not find any significant association between the nitrate content of public water supplies and gastrointestinal diseases. The evidence for nitrate as a cause of cancers of the digestive tract remains controversial [13].

Our findings are consistent with some previous studies. A case-control study conducted during three years in USA showed negligible associations of colon or rectum cancers with measures of nitrate in public water supplies, including average nitrate and the number of years with elevated average nitrate levels [14]. A cohort study in Finland found no association between dietary nitrite intake and stomach cancer. Another cohort study in the Netherlands found no association between stomach cancer risk and quintiles of water nitrate intake, which was determined from public supply levels and tap water intake [4].

On the other hand, some studies demonstrated positive correlations between water content of nitrate or nitrite and gastrointestinal diseases. Some human studies revealed an association between water nitrate and N-nitroso compounds formation in the gastrointestinal tract. Though, few individual-based studies exist on the relationship of N-nitroso compounds precursors with risk of gastrointestinal cancers. Most epidemiologic studies of drinking water nitrate and stomach cancer were ecologic and found mixed results [15].

An ecological study in 2001 investigated the association of gastric cancer mortality and nitrate content of drinking water and showed significant association. This study supports the hypothesis that the high level of nitrate in drinking water may have a role in the development of gastric cancer [16].

In our study, the nitrate concentration in the wells of one of the areas under study was higher in summer than in winter. In summer time, agricultural activities and water use increase; therefore sewage and drainage will result in more nitrate production.

These two wells near the spill area are considered as the main sources of the increase in agricultural land drainage effluent nitrate concentrations in summer. In our study, nitrate, nitrite, and TOC values were under the standard levels recommended by the WHO (50, 3, and 3 mg/L).

Some research studies suggest that increasing the allowed levels of nitrate in drinking water can be without risk to human health. Furthermore, some persons with high rates of endogenous formation of carcinogenic N-nitroso compounds may be vulnerable to the development of gastrointestinal cancers. Given the extensive experimental data suggesting a role for nitrate in the formation of carcinogenic N-nitroso compounds and the widespread exposure to nitrate in the population, limited epidemiologic data exist on addressing the possible association of nitrate in drinking water with cancer risk.

Nitrate levels in water supplies have been increasing at global level; therefore, additional population-based studies with well-characterized exposures are urgently needed to further our understanding of cancer risk associated with nitrate ingestion [17] Given the importance of water quality on human health [18], future longitudinal studies should determine the effects of exposure to water content of nitrite and nitrate on development of different gastrointestinal diseases. High levels of nitrate and nitrite in water can be because of using fertilizers; relevant environment and health organizations should control these issues.

### 4.1. Study Limitations

The major limitation of our investigation is its cross-sectional nature; thus a causal relationship cannot be inferred from our findings, and longitudinal studies are required to test for causality and the clinical importance of our findings.

## 5. Conclusion

Although we did not document significant association of nitrite, nitrate, and TOC content of water with gastrointestinal diseases, it should be considered that such health hazards may develop over time, and the quality of water content should be extensively controlled to prevent different health hazards.

## Figures and Tables

**Table 1:** Nitrate and nitrite levels in wells of the two areas under study in 2012.

Place of sampling	Nitrate concentrations in different months													
	February		March		April		June		July		Mean		Std. deviation	
	Nitrate (mg/L)	Nitrite (mg/L)	Nitrate (mg/L)	Nitrite (mg/L)	Nitrate (mg/L)	Nitrite (mg/L)	Nitrate (mg/L)	Nitrite (mg/L)	Nitrate (mg/L)	Nitrite (mg/L)	Nitrate	Nitrite	Nitrate	Nitrite
Area 1	30.5	0.02	31.45	0.02	31.63	0.015	32.2	0.017	32.2	0.013	31.738	0.0166	0.74405	0.0045
Area 2	22.7	0.026	23	0.01	22.86	0.014	22.86	0.016	24.4	0.022	23.033		0.59582	
